# Lung Ultrasound as a Bedside Tool for Assessment of Extravascular Lung Water in Critically Ill Head Injured Patients: An Observational Study

**DOI:** 10.5005/jp-journals-10071-23135

**Published:** 2019-03

**Authors:** Vasavi Gattupalli, Kajal Jain, Tanvir Samra

**Affiliations:** 1,2 Department of Anesthesia and Intensive Care, Postgraduate Institute of Medical Education and Research (PGIMER), Chandigarh, India

**Keywords:** Critical care, EVLW, Imaging, Ultrasonography

## Abstract

**Introduction:**

Extra vascular lung water (EVLW) is defined as the amount of fluid in the interstitial and alveolar spaces. Primary aim of this study was to assess EVLW using lung USG (B lines >3 per lung field) in critically ill head injured patients.

**Materials and methods:**

Intubated adult patients admitted in Trauma ICU with head injury (GCS 4-15) were assessed by daily chest X-ray and lung ultrasonography. Lung water content was graded based on the number of B lines per ICS with score ranging from 0-32 and categorized as low pulmonary fluid burden (0-10), moderate fluid burden (11-20) and high fluid burden (21-32).

**Results:**

140 critically ill head injured patients were assessed for eligibility and 20 excluded. Incidence of increased EVLW using lung USG was 61.66% (74/120) and the incidence reported using chest x ray was 40.83%(49/120) and the difference was statistically significant (*p* value <0.001). Increased EVLW significantly increased the duration of weaning, mechanical ventilation and ICU stay (p value <0.05). Significant association was observed between APACHE II, SAPS II and GCS at admission to ICU with presence of EVLW (*p* value ≤0.001). Mean delay in identification of EVLW by chest X-ray (CXR) compared to lung ultrasound was 1.42±0.76 days.

**Conclusion:**

Lung ultrasound is better than CXR for early detection of increased EVLW in critically ill head injured patients and has prognostic relevance as increased EVLW prolongs duration of mechanical ventilation and ICU stay.

**How to cite this article:**

Gattupalli V, Jain K, *et al*. Lung Ultrasound as a Bedside Tool for Assessment of Extravascular Lung Water in Critically Ill Head Injured Patients: An Observational Study. Indian J Crit Care Med 2019;23(3):131-134.

## INTRODUCTION

Extra vascular lung water (EVLW) is the amount of fluid in the interstitial and alveolar spaces and is normally <500 ml. Increase in lung water content of 75-100% from baseline leads to impairment in the physiological functions of the lung^1^. Chest radiography, computed tomography, central venous pressure (CVP) and PCWP are used to predict the pulmonary water content. But all these methods have their own drawbacks and limitations^2^.

USG examination of the lung is a bedside, non-invasive real time, radiation free, portable technique for detection of pulmonary abnormalities. Sensitivity and specificity of lung ultrasound in detecting alveolar interstitial syndrome is 93.4% and 93%, respectively^3^. Increase in water content of the lungs leads to visualization of multiple and diffuse B-lines. B-line is a well-defined, laser-like, hyperechoic comet-tail artifact arising from the pleural line.

The primary aim of this study was to assess EVLW using lung ultrasound in critically ill head injured patients. The secondary aim was to compare the diagnostic accuracy and time of identification of extra vascular lung water using lung ultrasound versus routine chest X-ray. We also observed the association of extra vascular lung water with weaning and duration of mechanical ventilation.

## MATERIALS AND METHODS

This prospective observational study was conducted in Trauma ICU from September 2015 to December 2016. Patients aged more than 16 years with head injury (GCS 4-15) and requiring ventilatory support were assessed for eligibility for enrollment in the study. Approval was obtained from institute ethics committee (INT/ IEC/2015/372) and written informed consent was obtained from the legal guardian of the critically ill patients. Patients with associated chest injury/ lung pathology and refusal of consent were excluded. Sample size calculation was done based on a previous study which reports the incidence of ALI/ARDS as 20-25% in patients with isolated TBI^4^. We chose a sample of 120 patients for an a error of 0.05 and ß error of 80%. Extra vascular lung water was assessed by daily serial chest x ray and lung ultrasonography.

### Chest X-Ray Ap View

Chest X-rays were taken daily in the ICU with the patient in the supine or semi recumbent position using portable X-ray machine (Allengers MobilX^DR^, Mobile Digital Radiography System) and was reviewed independently by two blinded observers for the evidence of pulmonary edema. Prominence of upper lobe vessels, Kerley lines B lines, sub pleural effusions, diffuse increase in parenchymal density (alveolar edema leading to batwing sign) and extensive peri hilar haziness were indicative of pulmonary edema.

### Lung Ultrasonography

Ultrasound machine and a 3-5MHz linear/ convex probe (SonoSite MicroMaxxPortable Ultrasound machine) was used for daily examinations in the ICU. Four inter costal spaces (ICS) were scanned in all enrolled patients in either supine/ semi recumbent position, which include third and sixth ICS on either side of sternum till mid clavicular line. Screen shots of every ICS examined were recorded, and two observers who were blinded to the details of the images analyzed them using the same scoring system. A total of four 10-s interval video files were obtained per patient and sent to two observers in two separate file sets for interpretation. The video files were independently interpreted by two observers who were blinded from the patient data, to verify the number of B-lines. If the numbers of B-lines counted by two observers were not concordant, the consensus number was used. Increased EVLW was defined as >3 B lines per lung field and the lung water content was then graded based on the number of single and confluent B lines per ICS with score ranging from 0-32 (Table 1)^5^.

CXR and lung ultrasound was done every 24 hrs till the patient was on mechanical ventilatory support (eVent medical, lake forest, California, USA). Minute ventilation was adjusted to establish a PaCO_2_ within a range of 30-35mm Hg. Heat and moisture filters were used for humidification and protective purposes. Patients were sedated using midazolam/morphine infusion as per ICU protocol.

**Table 1:** Lung ultrasound scoring system

*Ultrasound findings*	*Score*
No B line/ICS	0
One B line/ICS	1
Two B lines/ICS	2
Three B lines/ICS	3
Four B lines/ICS	4
Five B lines/ICS	5
Confluent B lines >50% ICS	6
Confluent B lines >75% ICS	7
Confluent B lines 100% ICS	8

Total score was obtained after adding the independent values of right and left upper and lower lung fields (a total of 4 values). Pulmonary fluid burden graded as low (score 0-10), moderate (score 11-20) and high (score 21-32).

**Table 2:** Demographic data and overall patient characteristics (N=120)

*Parameters*	
Age (years)	36.88±14.82
Gender (Male: Female)^*^	94:26
GCS at admission to ICU	8.35±2.72
Marshall score	3.03±0.90
APACHE II score	11.63±4.22
SAPS II score	25.48±10.7
Duration of mechanical ventilation (days)	7.93±7.62
Duration of ICU stay (days)	9.23±8.15
Mortality^*^	10

Values expressed as Mean±SD

*Values expressed as number

The statistical analysis was carried out using Statistical Package for Social Sciences (SPSS Inc, Chicago, version 20.0 for windows). Normality of quantitative data was checked by Kolmogorov- Smirnov tests of normality. Mann-Whitney U test was applied for statistical analysis of skewed data. For normally distributed data, t-test was applied for comparison between two groups. Qualitative or categorical variables are described as frequencies and proportions. Proportions are compared using Chi square or Fischer exact test whichever was applicable. A *p* value of <0.05 was considered statistically significant.

Total duration of mechanical ventilation, total duration of ICU stay, APACHEII, SAPSII, in patients with and without EVLW was compared using two tailed students t test. Mortality rate between the two groups was compared using Chi square test.

## RESULTS

140 patients were assessed for eligibility out of which 120 fulfilled the inclusion criteria and were enrolled in the study. Of these, 20 patients had to be excluded from the analysis as 12 patients had mortality within 24 hours and 8 had transmissible infectious diseases. Demographic and clinical characteristics of patients are summarized in Table 2.

Increased EVLW using lung USG was reported in 61.66% of the patients during stay in ICU (74/120) but the incidence reported using chest x ray during the same period was only 40.83% (49/120). This difference in incidence reported by two different diagnostic modalities was statistically significant (*p* value <0.001). Pulmonary fluid burden was graded using lung USG scoring system as low in 13.51% (score 0-10), moderate in 60.81% (score 11-20) and high in % (score 21-32) of patients.

Increased EVLW at the time of admission to ICU was observed in 32 (43.24%) patients using lung USG but reported in only 6 (8.11%) patients using chest X-ray and this difference in incidence reported by two different diagnostic modalities was statistically significant (*p* value <0.001). Follow-up lung ultrasound done daily added 42 patients to the existing 32 whereas follow up daily CXR added 43 patients to the existing 6. Mean delay in detection of EVLW on CXR after detection on ultrasound was calculated as 1.42±0.767 (Mean±SD) days.

Comparison of demographic data and characteristics of patients with and without evidence of EVLW (Table 3). Statistically significant difference was noted in the GCS (at admission), APACHE II and SAPS II of patients with and without the presence of EVLW (p<0.001). S. Albumin levels in patients with presence of EVLW was 3.46±0.48 (Mean ± SD) and in patients without any excess EVLW was 3.87±0.45 (Mean±SD). Pao2/Fio2 ratio was in patients with and without EVLW, 419.43±62.23 and 293.62±82.89, respectively (Mean±SD).

**Table 3:** Comparison of demographic data and clinical characteristics of patients with and without presence of EVLW

	*EVLW(+) N=74*	*EVLW(-) N=46*	*p value*
Age (years)	38.24±14.93	34.6±14.55	0.201
Marshall score	3.14±0.849	2.85±0.96	0.09
GCS (on admission)	7.73±2.68	9.35±2.52	0.001
APACHE II score	12.86±3.50	9.65±4.56	<0.001
SAPS II score	28.3±10.12	20.96±10.40	<0.001
S. Albumin < 3.5^#^	37	10	< 0.001
Pao2/Fio2 <300^#^	49	0	0.001
Duration of MV(days)	10.01±9.02	4.57±1.69	0.001
Duration of ICU stay(days)	11.38±9.71	5.78±1.75	0.01
Duration of weaning (days)	10.22±9.46	4.46±1.57	0.002

Data compared using t test, expressed as mean±SD. MV: Mechanical ventilation *p* Value <0.05 significant.

# Values expressed as number.

**Table 4:** Result of weaning trial in patients with and without presence of EVLW

*Category*	*EVLW(+) N=74*	*EVLW(-) N=46*	*Total*	*p value*
Difficult	16	0	16	<0.001
Simple	48	46	94	
Not tried	10	0	10	
Total	74	46	120	

Values expressed as number. Data compared using Chi square test. *P* value <0.05 significant

Success of weaning trial in patients with and without excessive lung water content are summarized in Table 4. Patients who could be weaned successfully on the first attempt were considered to be in the category of simple weaning. Patients who failed initial weaning and required upto three trials or as long as 7 days from the first trial to achieve successful weaning were considered to be in the category of difficult weaning.

In our study, 84 patients (n=84/120, 70%) received conservative management; decongestants like mannitol and/or frusemide and the remaining 36 patients (n=36/120, 30%) received surgical management. There was no significant difference in the prevalence of EVLW in patients managed conservatively or surgically i.e., 60.71% (n=51/84) and 63.89% respectively.

## DISCUSSION

In our study, the use of lung ultrasound showed evidence of increased EVLW in 61.66% (74) of patients during the study period. Incidence of pulmonary edema in isolated head injury in previously published studies has been reported to be 50-85%^6,7^. In our study the use of chest X-ray reported a lower incidence of 40.83% and the difference in mean duration of identification of lung water by lung ultrasound and chest X-ray was 1.42±0.767 days (Mean±SD). Thus, use of USG facilitated greater diagnostic accuracy and detection at an early stage. Bedside Lung Ultrasound in Emergency (BLUE protocol) for evaluation of patients with acute respiratory failure have reported sensitivity and specificity of lung ultrasound (based comet tail artifacts) in diagnosing pulmonary edema to be 97% and 95%, respectively^8^.

Various diagnostic modalities like Chest X-ray, Computed Tomography, Nuclear Magnetic Resonance imaging, Positron Emission Tomography, Indicator dilution method have been used for detection of EVLW. Radiation hazards, requirement of huge infrastructure, low specificity and high inter observer variability are the limitations with the above mentioned modalities. Lung USG is a non-invasive, radiation-free and bedside portable tool for detection of pulmonary edema. Scanning of the lung using ultrasound can be done using several approaches; 28 sector, 8 sector and 4 sector. The 4 sector protocol has been validated (excellent correlation with the results of trans pulmonary thermo dilution) for detection of EVLWI and so we used this protocol in our study^5^.

The increased lung water content is due to neurogenic pulmonary edema which is due to surge in adrenergic responses following head injury and activation of mediators like IL-6 which cause transudation and accumulation of fluid in the interlobular septa. Increase in greater than 50% of interstitial fluid exceeds the resorptive capacity of the lymphatic system and leads to alveolar edema which causes impairment in gas exchange and clinical signs^9,10^.

Our study highlights that patients with low GCS, low S. Albumin (< 3.5^#^), low Pao2/Fio2 (<300) and greater APACHE II and SAPS II have significantly higher incidence of interstitial pulmonary edema. Bratton et al. have reported lower Pao2/Fio2 in patients with isolated TBI associated with higher global severity of head injury (low GCS)^11^. Bilotta et al. have reported reduction in Pao2/Fio2 in patients with B lines in neurocritical care patients^12^. In our study also, we postulate that the TBI caused increased EVLW (neurogenic pulmonary edema) and lower Pao2/Fio2. Lower albumin facilitates transudation of fluids and increases incidence of pulmonary edema. Thus, higher vigilance is needed for detection and management of alveolar interstitial syndrome (AIS) at an early stage in this subset of patients. Early management can be in the form of fluid balance, diuretics, morphine and use of high PEEP.

Our study also showed a significant increase in duration of weaning, duration of mechanical ventilation, and duration of ICU stay in patients with presence of EVLW (*p*value <0.05). Holland et al. also reported increase in duration of mechanical ventilation in patients with isolated TBI and ALI^4^. EVLW correlated well with survival (i.e., nonsurvivors had significantly higher EVLW values than survivors) and was an independent predictor of prognosis in critically ill patients in a study conducted by Sakka et al.^13^ Thus, early detection and prompt management of presence of EVLW is of paramount importance.

Limitation of our study was that other contributing factors could have led to the development of AIS; neurogenic edema cannot be labeled as the sole causative agent. However, the fluid balance was similar in both the groups but there was higher requirement of PEEP in patients with EVLW to attain adequate Pao2 levels on blood gas analysis compared to patients without EVLW. CT scan was not considered in our study because of lack of availability of bedside CT in ICU. It could have given better knowledge regarding sensitivity and specificity of lung ultrasound. Further studies are required to investigate the various interventional approaches adopted for management of increased lung water content.

## CONCLUSION

Sixty percent of patients with head injury have increased lung water content. We observed greater diagnostic accuracy and earlier identification of EVLW using lung ultrasound compared to chest x ray. Patients with low GCS, low S. Albumin (< 3.5^#^), low Pao2/Fio2 (<300) and greater APACHE II and SAPS II had significantly higher incidence of interstitial pulmonary edema. Early detection of EVLW is needed to minimize the duration of weaning, duration of mechanical ventilation and duration of ICU stay.
